# The complete chloroplast genome sequence of *Rehmannia glutinosa* (Gaertn.) DC. *Wild.* (*Rehmannia*)

**DOI:** 10.1080/23802359.2021.1881837

**Published:** 2021-03-11

**Authors:** Hao Yang, Yohei Sasaki, Conglong Lian, Lili Wang, Fei Zhang, Xueyu Zhang, Suiqing Chen

**Affiliations:** a Pharmacy College of Henan University of Chinese Medicine, Zhengzhou, China; b College of Health and Medicine, Kanazawa University, Kanazawa, Japan

**Keywords:** *Rehmannia glutinosa* Wild, chloroplast genome, phylogeny

## Abstract

In this study, we constructed and annotated a complete circular chloroplast genome of wild *R. glutinosa*. The chloroplast genome of wild *R. glutinosa* is 153,678 bp in length, including two inverted repeat (IR) regions of 25,759 bp, separated by a large single copy (LSC) region of 84,544 bp and a small single copy (SSC) region of 17,616 bp. The genome contains 149 genes, including 104 protein-coding genes, 37 tRNA genes, and eight rRNA genes. Neighbor-joining method phylogenomic analysis showed that wild *R. glutinosa* formed a monophyletic group, and was sister to other groups of *R. glutinosa*.

Wild *Rehmannia glutinosa* (Gaertn.) DC. is a perennial herb of the *Scrophulariaceae* family. The wild and cultivated species of *R. glutinosa* belong to the same plant in classification, and the morphological characteristics are very similar, but the underground root tuber size difference is very obvious (Li et al. 2013). Therefore, different scholars have different nomenclature and classification, and both Chinese Flora and Chinese Pharmacopoeia unify the scientific name of Rehmanniae radix into *R. glutinosa* from the perspective of large species (Lou et al. 1995). Xie believes that from the viewpoint of paying attention to authentic medicinal materials in the development of traditional Chinese medicine, it is believed that the thickness of underground parts of wild *R. glutinosa* and cultivated *R. glutinosa* is different, and the quality of medicinal materials is different. In order to ensure the correct supply of drugs according to the name of drugs, the scientific names of the two are still appropriate to be applied separately (Xie 1990).

In recent years, DNA barcoding has developed into a powerful tool for species identification. Compared with common DNA barcode short fragments, the whole chloroplast genome contains more abundant mutation sites and identification efficiency is high (Jiang et al. 2020). Therefore, in this study, we assembled the complete chloroplast genome of wild *R. glutinosa* based on next generation sequencing technology, analyzed the basic structure of the chloroplast genome of wild *R. glutinosa* revealed the phylogenetic relationship between wild and cultivated *R. glutinosa* by constructing a phylogenetic tree, provided a molecular basis for the classification and nomenclature of wild and cultivated *R. glutinosa* and laid the foundation for the excavation of excellent genes of wild *R. glutinosa*.

Fresh leaves of wild *R. glutinosa* were collected from Wanxianshan (113°61′78.22″E, 35°72′27.10″N) in Xinxiang City, Henan Province, China and stored in the herbarium of Henan University of Traditional Chinese Medicine with voucher specimen number: HZYYHC15. Total genomic DNA was extracted using a Rapid Plant Genomic DNA Isolation extraction kit.

This experiment adopts the Illumina HiSeq PE150 platform, a 300 bp (insertion size) double-ended library was constructed by splicing DNA. First, the quality of the original sequencing data was evaluated by FastQC, then the quality of the sequencing data with low quality was cut by Trimmomatic to obtain relatively accurate and effective data. SPAdes 3.13.1 was used to assemble the filtered sequencing data and GapFiller was used to fill gaps in assembled contigs (Bankevich et al. 2012). PrlnSeS-G was used for sequence correction. Plann is used for initial annotation (Huang and Cronk 2015). The complete annotated chloroplast genomic sequence annotated had been submitted to GenBank under the accession number of MW007380 for wild *R. glutinosa*.

The structure of chloroplast genome of wild *R. glutinosa* was circular, and the size was 153,678 bp, including two inverted repeat (IR) regions of 25,759 bp, separated by a large single copy (LSC) region of 84,544 bp and a small single copy (SSC) region of 17,616 bp. The whole chloroplast genome GC content is 37.91%. There are 149 genes in the chloroplast genome of wild *R. glutinosa* including 104 protein-coding genes, 37 tRNA genes, and eight rRNA genes.

We selected 15 chloroplast genome sequences of *R. glutinosa* and *Scrophularia dentata* Royle ex Benth. (*Scrophulariaceae*) as outgroups and constructed a phylogenetic tree using the ML (maximum likelihood) method (bootstrap: 1000) embedded in MEGA software (Xia et al. 2016), the results showed that *R. glutinosa* was clustered separately in the mainland of *Rehmannia* and wild and cultivated *R. glutinosa* were sister clades. Among them, these samples (KX636157\NC034308) were transplanted from wild to cultivated (Zeng et al. 2017). The phylogenetic tree demonstrated the domestication process from wild to cultivated (Figure 1). Xie’s view is also supported at the chloroplast genome level to classify and name wild and cultivated *R. glutinosa*.

**Figure 1.**
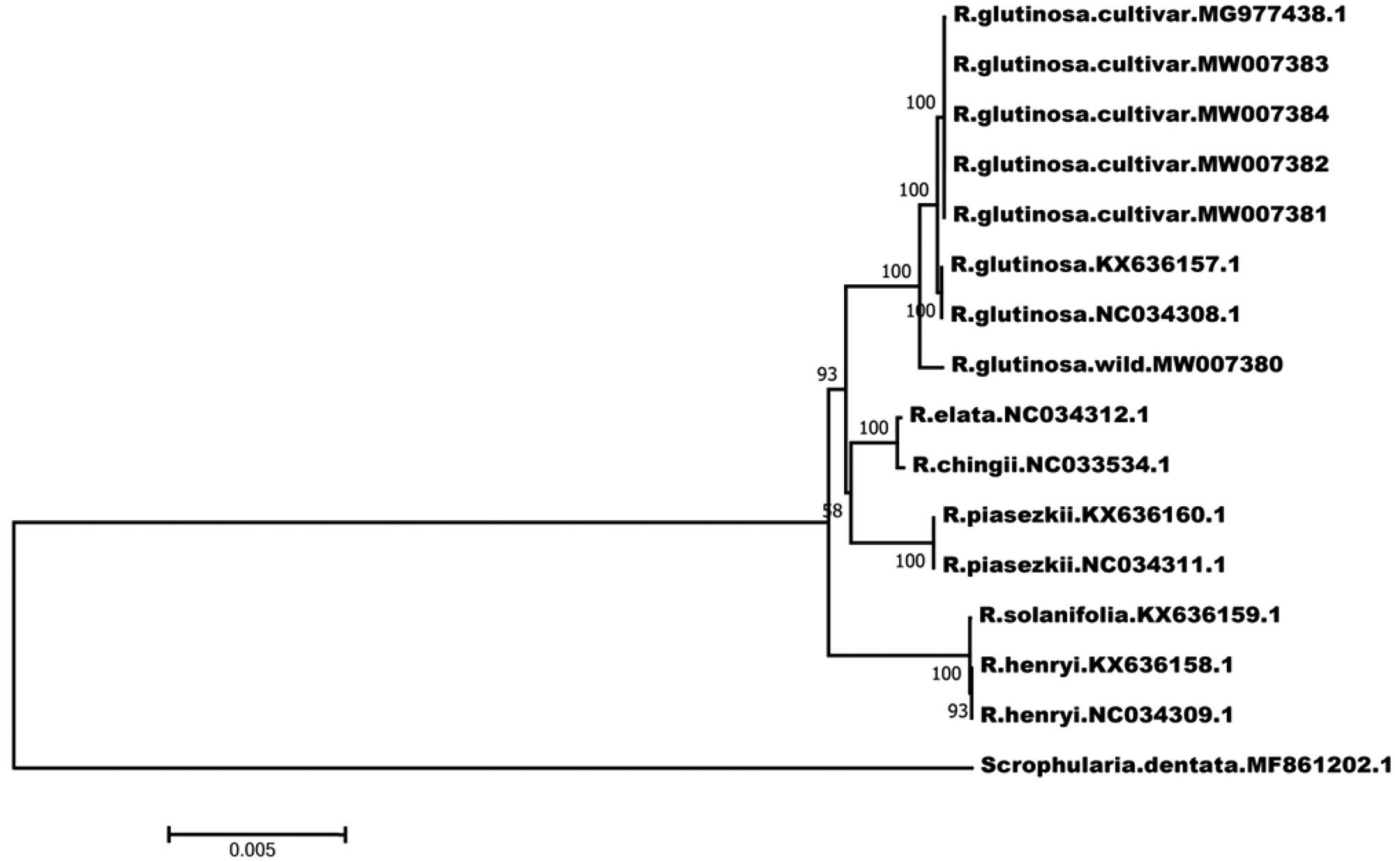
The best ML phylogenetic tree (bootstrap: 1000) from 15 *Rehmannia* complete chloroplast sequences.

## Data Availability

The genome sequence data that support the findings of this study are openly available in GenBank of NCBI at https://www.ncbi.nlm.nih.gov/nuccore/MW007380.1/ under the accession no. MW007380. The associated BioProject, SRA, and Bio-Sample numbers are PRJNA682578, SRR13201823, and SAMN16992822, respectively.

## References

[CIT0001] Bankevich A, Nurk S, Antipov D, Gurevich AA, Dvorkin M, Kulikov AS, Lesin VM, Nikolenko SI, Pham S, Prjibelski AD, et al. 2012. SPAdes: a new genome assembly algorithm and its applications to single-cell sequencing. J Comput Biol. 19(5):455–477. 22506599 10.1089/cmb.2012.0021 PMC3342519

[CIT0002] Huang DI, Cronk Q. 2015. Plann: a command-line application for annotating plastome sequences. Appl Plant Sci. 3(8):1500026. 10.3732/apps.1500026 PMC4542940 26312193

[CIT0003] Jiang W, Guo M, Pang X. 2020. Application of chloroplast genome in identification and phylogenetic analysis of medicinal plants. World Tradit Chin Med. 15:702–708.

[CIT0004] Li X-E, Sun P, Qi J-J, Zhou L-L, Wang S-H. 2013. Changes of hormones in cultivars and wild-type varieties of *Rehmannia glutinosa* Libosch. J Crops. 39(7):1276–1283.

[CIT0005] Lou Z-c, Qin B. 1995. Study on the classification and quality of common Chinese medicinal materials – Volume 3 – Northern Edition. Beijing: Beijing Medical University, China Union Medical University Press.

[CIT0006] Xia Z, Wang L-j, Huang Y, Li H-m, Zhang H-r, Gao Z-m. 2016. Identification of DNA barcoding in plants of Rehmannia Libosch. ex Fisch. et Mey. and origin of cultivated *Rehmannia glutinosa*. Chin Herb Med. 47:648–654.

[CIT0007] Xie Z-w. 1990. Discussion on medicinal materials of Taoism. J Chin Med. 10:43–46.

[CIT0008] Zeng S, Zhou T, Han K, Yang Y, Zhao J, Liu ZL. 2017. The complete chloroplast genome sequences of six Rehmannia species. Genes (Basel). 8(3):103. 10.3390/genes8030103 PMC5368707 28294981

